# Immersion Freezing Efficiency of ZnAl_2_O_4_ and MgAl_2_O_4_ Spinels, ZnO, and MgO: The Role of Oxygen Vacancies

**DOI:** 10.1021/acs.jpca.5c06327

**Published:** 2026-02-09

**Authors:** Ryan Mitch, Ayat Tassanov, Brendan P. Troesch, Mikyung Hwang, Nathan Baumann, Konstantinos Alexopoulos, James M. Hodges, Miriam Arak Freedman

**Affiliations:** † Department of Chemistry, 8082 The Pennsylvania State University, University Park, Pennsylvania 16802, United States; ‡ Department of Chemical Engineering, 8082 The Pennsylvania State University, University Park, Pennsylvania 16802, United States; § Department of Meteorology and Atmospheric Sciences, The Pennsylvania State University, University Park, Pennsylvania 16802, United States

## Abstract

Aerosol particles that catalyze ice nucleation alter the optical properties and precipitation cycles of clouds. Although mineral dust aerosol particles containing metal oxides are susceptible to the formation of oxygen vacancies (*V*_O_) on their surfaces, the impact of these defects on ice nucleation activity has not been addressed. To investigate the impact of *V*_O_ sites, we conducted a droplet immersion freezing assay on zinc aluminate (ZnAl_2_O_4_) and magnesium aluminate (MgAl_2_O_4_) spinels annealed under air, nitrogen, and oxygen atmospheres. We observe that samples annealed under nitrogen promote ice nucleation at warmer temperatures compared to those treated in oxidizing atmospheres, with the effect being most pronounced for ZnAl_2_O_4_. To further understand these results, we investigated the immersion freezing of zinc oxide (ZnO) and magnesium oxide (MgO). Here, we observe that ZnO nucleates ice at substantially warmer temperatures than MgO after annealing under nitrogen. We hypothesize that the trends in ice nucleation activity are due to the varying concentrations of *V*_O_ that form during the annealing process on the oxide surfaces, which tend to be higher in the absence of O_2_. Density functional theory (DFT) calculations support our hypothesis, indicating that *V*_O_ is more stable on the surfaces of the Zn-containing oxides. The study suggests that oxygen vacancies, which are common defects on metal oxide surfaces that affect their adsorption and catalytic properties, can influence the efficiency with which mineral dust aerosol particles activate ice formation and affect cloud radiative forcing.

## Graphical abstract

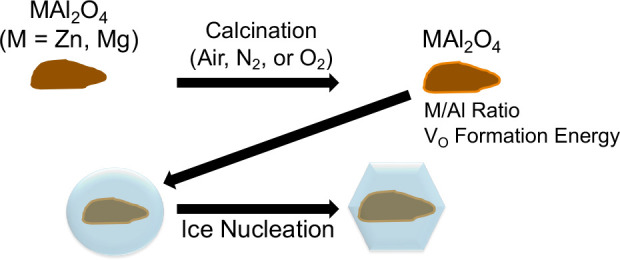

## Introduction

The formation of ice from other thermodynamically stable phases of water is a fundamental phase transformation which limits the long-term stability and longevity of tissues and cells of living organisms,^1^ and causes plants to sustain frost damage due to the presence of ice-nucleating bacteria.^2^ From an atmospheric chemistry perspective, ice formation affects the microphysical properties of clouds as well as their radiative forcing.^3,5^ Specifically, increasing concentrations of ice-nucleating particles can facilitate the release of precipitation from mixed-phase clouds, shorten their lifetime, and thereby impact albedo.^3,4^

Ice nucleation proceeds either homogeneously or heterogeneously. Homogeneous ice nucleation, which requires water or aqueous solution, occurs at temperatures less than or equal to approximately −38 °C.^5^ In contrast, heterogeneous ice nucleation is facilitated by a stabilizing surface and theoretically takes place at any temperature at or below 0 °C.^6^ Several different modes of heterogeneous ice nucleation occur.^6^ In immersion freezing, supercooled aqueous droplets surround one or many solid, insoluble ice-nucleating particles (INP).^7^ In contact freezing, supercooled aqueous droplets collide with INP, initiating the freezing process.^7^ In condensation nucleation, soluble material on INP deliquesces and subsequently freezes once the activity of the aqueous solution becomes sufficiently high.^7^ Ice nuclei can form from water vapor that is supersaturated with respect to ice via the deposition mode, which is observable in a laboratory environment, but has been proposed to instead result from homogeneous or heterogeneous freezing initiated in pores or cracks in a process termed pore condensation freezing.^5,8^ Some of these modes are not differentiable experimentally depending on the conditions. Among the primary ice production pathways, immersion freezing is suggested to predominate during mixed-phase cloud glaciation, while deposition freezing/pore condensation freezing may be most relevant to cirrus cloud formation.^8^

The chemical composition of mineral dust particles and chemical adsorbates influence their ice nucleation activity. Zolles et al. discussed how the ice nucleation activity of feldspars is affected by the electronic effects of charge-balancing cations. They hypothesize that cations associated with feldspar surfaces immersed in aqueous solution interact with neighboring water molecules, and cation sizes and charge densities dictate their kosmotropic or chaotropic (water ordering/disordering) behaviors, impacting ice nucleation in the vicinity of feldspar surfaces.^9^ Both calcium and sodium cations are chaotropic and thus disrupt the hydrogen-bonding network of water molecules, while potassium cations are kosmotropic and weakly interact with water. Thus, water molecules are more immobilized in the hydration shells of chaotropic charge-balancing cations compared to water molecules surrounding potassium cations, and the solution conditions become less conducive to ice nucleation for the former species compared to the latter.^9^ Therefore, ice nucleation of the calcium- and sodium-containing plagioclase feldspars is suppressed compared to potassium-rich feldspars.^9^ Jin et al. explored the effect of ion exchange on the ice nucleation activity of potassium-rich mica immersed in a series of alkali metal chloride salt solutions.^10^ They correlated warmer ice nucleation temperatures with increases in the size of the metal cation, following the same line of reasoning as Zolles et al. Marak et al. observed that sodium adsorbates on the surface of ZSM-5 zeolites enhance their ice nucleation activity as compared to ammonium adsorbates, and hypothesized that ammonium cations block nucleation sites during adsorption to the zeolite surface and/or hydrogen bond strongly to surrounding water molecules, forcing their assembly into unfavorable orientations for ice formation.^11^ In addition, they found that a higher Al/Si ratio at the zeolite surface favors ice nucleation at warmer temperatures perhaps due to the presence of more Brønsted acid sites.^11^ Whale highlighted the competition between the chemical characteristics and colligative properties of ammonium ions, observing that introducing dilute concentrations enhances the ice nucleation activity of feldspars, possibly by aiding the hydrogen bonding of neighboring water molecules, while higher concentrations disfavor it as freezing point depression predominates.^12^

Trace elements in mineral dust particles also affect their ice nucleation activity. Welti et al. correlate warmer ice nucleation temperatures with an increase in the Rb/Sr ratio of plagioclase and potassium-rich feldspars, suggesting that both Rb^+^ and Sr^2+^ cations exchange with intrinsic K^+^/Na^+^ and Ca^2+^ cations to augment the kosmotropic (water-ordering) character.^13^ However, they note that the trace impurity concentrations necessary to alter the ice nucleation activity must be quantified to investigate this relationship more thoroughly.^13^ Cziczo et al. observe that PbO embedded in kaolinite particles lowers the supersaturation at which the onset of ice nucleation is observed due to the high lattice match between PbO and ice *I*_h_.^14^ Although the aforementioned studies highlight the influence of structural features on the ice nucleation activity of mineral dust particles, they focus primarily on the chemical characteristics of inorganic metal ions embedded in the mineral structural frameworks and thus do not address the contributions to ice nucleation activity from other common structural features or deficiencies, including *V*_O_ sites on the surfaces of metal oxides.

The nature and concentration of surface V_O_ play an important role in heterogeneous catalysis and influence the adsorption of small molecules on metal oxide surfaces,^15–17^ including spinel-type oxides.^18,19^ Similar to feldspars and aluminosilicate clay minerals, spinel-type minerals can accommodate a wide range of metal cations and exhibit order–disorder behavior, making them an interesting model system for investigating the role of oxygen vacancy sites on ice nucleation activity. Spinels are used in numerous catalytic, optical, magnetic, and electrochemical applications due to their tunable surface chemistry.^20^ For applications in catalysis, well-defined metal oxides are often prepared using hydrothermal methods, where metal precursors are reacted in an aqueous solution under high pressure.^20–23^ The surface chemistry of the oxide products can then be tailored through postsynthesis annealing under various atmospheres.

Since mineral dust aerosol particles contain oxides, *V*_O_ sites may be present on their surfaces. Therefore, to gain insight into the role that *V*_O_ sites play in the ice nucleation of mineral dust aerosol particles, we prepared spinel-type ZnAl_2_O_4_ and MgAl_2_O_4_ oxides using a hydrothermal protocol. The spinel products were annealed at 900 °C under air, nitrogen, and oxygen atmospheres to modulate their surface chemistry. Immersion freezing experiments showed that spinels annealed under nitrogen exhibit a higher propensity to nucleate ice when compared to those treated under air or oxygen. We hypothesize that this difference in ice nucleation activity is due to higher concentrations of *V*_O_ defects on the surface of the spinel substrates, which is expected when annealing at high temperatures in inert atmospheres.^24^ Regardless of the annealing conditions, ice nucleation was observed to occur at warmer temperatures in ZnAl_2_O_4_ compared to MgAl_2_O_4_. This difference can be attributed to both cation speciation and a greater concentration of *V*_O_ defects produced during annealing of the Zn-containing spinel, as supported by density functional theory (DFT) calculations. We note that surface *V*_O_ could influence ice nucleation in multiple ways, including indirectly by affecting the concentration of surface-bound hydroxyl species. To further explore this connection, we also probed the ice nucleation activity of the binary metal oxides ZnO and MgO, which were annealed under the same conditions. Again, the metal oxides annealed under nitrogen exhibit a higher propensity to nucleate ice compared to those treated under air or oxygen, although direct comparison of the binary oxides is nontrivial since they have different crystal structures. We discuss the implications of our results for the study of atmospheric ice nucleation.

## Experimental Section

### Materials

The compounds: zinc nitrate hexahydrate (Zn­(NO_3_)_2_·6H_2_O, 98% purity), magnesium nitrate hexahydrate (Mg­(NO_3_)_2_·6H_2_O, 99% purity), aluminum nitrate nonahydrate (Al­(NO_3_)_3_·9H_2_O, 99% purity), magnesium oxide (MgO, 99% purity), zinc oxide (ZnO, 99% purity), and ammonium hydroxide solution (NH_3_ in water, 25 wt %) were purchased from Sigma-Aldrich and used without any pretreatment.

### Synthesis

MgAl_2_O_4_ and ZnAl_2_O_4_ were synthesized by using a hydrothermal method. The corresponding nitrate hydrates (Mg­(NO_3_)_2_·6H_2_O, Zn­(NO_3_)_2_·6H_2_O) were mixed with Al­(NO_3_)_3_·9H_2_O in 5:10 mmol ratios to synthesize MgAl_2_O_4_ and ZnAl_2_O_4_. The powders were placed in a beaker with a magnetic stir bar, and 49 mL of deionized water was added to dissolve the salts. The solutions were stirred for 15 min, followed by the addition of 10 mL of ammonium hydroxide solution (25 wt %). A white gel formed immediately upon adding ammonia, and the mixture was stirred for an additional 30 min to homogenize. The pH of the mixtures was approximately 10.5–11. The resulting gels were transferred to a Teflon-lined stainless-steel autoclave and heated at 225 °C for 24 h. After being naturally cooled, the samples were centrifuged twice with deionized water and once with acetone to remove the supernatant. The white gels were then dried overnight at 80 °C.

### Annealing Experiments in Different Atmospheres

Synthesized MgAl_2_O_4_, ZnAl_2_O_4_, MgO, and ZnO were calcined (annealed) in three different atmospheres at 900 °C. For air annealing, the samples were placed in alumina boats and heated in a programmable muffle furnace at a rate of 100 °C/h, then soaked at 900 °C for 5 h, followed by radiative cooling to room temperature. For O_2_ and N_2_ annealing, the samples were placed in alumina boats inside a tube furnace with a continuous flow of the respective gas, then heated to 900 °C where they were soaked for 5 h followed by radiative cooling.

### X-ray Diffraction

All samples were pulverized into a fine powder by using an agate mortar and pestle. X-ray diffraction (XRD) data were obtained on a benchtop BRUKER D2 phaser diffractometer with Cu Kα radiation (λ = 1.5406 Å).

### Scanning Electron Microscopy (SEM)

The morphology and chemical homogeneity of spinel oxides were characterized by using a Verios G4 scanning electron microscope. The current used was 0.80 nA, with an accelerating voltage of 5 kV, and the working distance was 5.1 mm.

### Surface Area

Surface area measurements were performed on a Micromeritics 3Flex instrument at −196 °C. Before measurements, samples were degassed on a Micromeritics external VacPrep station at a rate of 2.7 °C h^–1^ and held at 300 °C overnight before cooling. Surface area was calculated by using a multipoint Brunauer–Emmett–Teller (BET) method.

### Immersion Freezing

After hydrothermal synthesis and annealing, the spinels and metal oxides underwent no further processing or chemical treatment, and sample preparation for ice nucleation experiments occurred within 1 to 2 days after each synthesis. Each sample was suspended in UHPLC-MS-grade water (Thermo Scientific) at a concentration of 0.4 wt % and sonicated for 20 min before each trial to ensure homogeneous dispersion of the solid particles. Following sonication, 2-μL droplets of each colloidal suspension were dispensed onto a clean and dry hydrophobic siliconized glass slide (Hampton Research), with a total of 3 trials performed and 108 droplets analyzed per material type. Each slide was individually placed inside a custom-built immersion freezing chamber with a N_2_ purge flow, the operating principles of which have been previously described by Alstadt et al. (Figure S1).^25^ Droplet freezing was visually detected for each trial by using a charge-coupling device (CCD) camera installed above the chamber, and a lamp was placed near the camera lens to illuminate the interior of the chamber, increasing the visibility of the droplet freezing tests. Images of each droplet freezing assay were automatically acquired every 0.5 °C at an average cooling rate of −3 °C/min using LabView. Freezing events were identified by sudden increases in the droplet opacity, indicating the formation of ice from liquid water.

To analyze the data collected during the immersion freezing assay, the frozen droplets from each trial at each recorded temperature within the 0.5 °C supercooling interval limit were counted, yielding the temperature-dependent frozen fraction, or *F*(*T*). Frozen fraction values were used to calculate the ice nucleation active site density per unit volume, or *K*(*T*), according to eq 1,

1 $$K\left(T\right)=\frac{\ln\left(1-F\left(T\right)\right)}{V_{drop}}$$

where *V*_drop_ is the volume of an individual water droplet (mL).^26,27^ The values for *K*(*T*) are subsequently converted to the number of active sites per surface area, *n*_s_, using eq 2

2 $$n_{s}=K\left(T\right)\times {\left(C\times SA_{BET}\right)}^{-1}$$

where *C* is the concentration of the prepared colloidal suspension (g/mL) and SA_BET_ is the Brunauer–Emmett–Teller surface area of the sample (m^2^/g). Error bars in the frozen fraction figures represent ±one standard deviation of the frozen fraction between the three trials, while one-sided error bars are displayed for *n*_s_ when appropriate, as some lower error bars cannot be displayed due to the use of a logarithmic scale.

### XPS Analysis

XPS experiments were performed using a Physical Electronics VersaProbe III instrument equipped with a monochromatic Al Kα X-ray source (hν = 1,486.6 eV) as well as achromatic Mg Kα (1253.6 eV) and Zr L (2042.4 eV), and a concentric hemispherical analyzer. Charge neutralization was performed using both low-energy electrons (<5 eV) and argon ions. The binding energy axis was calibrated using sputter-cleaned Cu (Cu 2p_3/2_ = 932.62 eV, Cu 3p_3/2_ = 75.1 eV) and Au foils (Au 4f_7/2_ = 83.96 eV). Measurements were made at a takeoff angle of 70° with respect to the sample surface plane. This resulted in a typical sampling depth of 2–5 nm (Mg), 3–6 nm (Al), and 5–7 nm (Zr), where 95% of the signal originated from this depth or shallower. Quantification was done using instrumental relative sensitivity factors (RSFs) that account for the X-ray cross-section and inelastic mean free path of the electrons. On homogeneous samples, major elements (>5 atom %) tend to have standard deviations of <3%, while minor elements can be significantly higher. The analysis sample size was ∼200 μm in diameter.

### DFT Analysis

DFT was implemented with the Vienna ab initio simulation program (VASP) using the Perdew–Burke–Ernzerhof (PBE) exchange-correlation functional.^28,29^ The strongly oscillating wave functions of core electrons were represented by the projector-augmented wave (PAW) method.^30^ Convergence during geometric optimization was determined when the forces on the atoms reached less than 0.05 eV/Å. The self-consistent field tolerance was set to 10^–5^ eV. A plane-wave basis set cutoff energy and the Monkhorst–Pack *k*-point mesh values are listed in Table S1.^31,32^ All calculations were spin-polarized. Considered valence configurations for each atom type are Zn 3d^10^ 4s^2^, Mg 3s^2^, Al 3s^2^ 3p^1^, and O 2s^2^ 2p^4^. To correct the self-interaction error from strongly correlated d-orbital electrons on Zn, the Hubbard’s *U* parameter of 3 eV was applied.^33^ Metal oxide structures were obtained from the American Mineralogist Crystal Structure Database.^34^ The crystal structure was optimized and then slab models were built using the Amsterdam Modeling Suite (AMS) considering the low-index facets as listed in Table S1.^35^ The surface model consisted of four layers where the number of layers converged the surface energy within 0.01 J/m^2^. The bottom two layers were fixed to simulate the underlying bulk phase, relaxing the remaining two layers during structural optimization. To minimize dipole interactions between periodic repeats, a vacuum space of 15 Å in the *z*-direction normal to the surface was included. A Wulff construction method was used to quantify the exposed ratio of low-index facets of each metal oxide.^36,37^

**Table 1:** *T*_10_, *T*_50_, and *T*_90_ Values, as well as Slopes, for ZnAl_2_O_4_ and MgAl_2_O_4_ Spinels following N_2_ and O_2_ Treatment

Sample	*T*_10_ (°C)	*T*_50_ (°C)	*T*_90_ (°C)	Slope (% frozen/°C)
ZnAl_2_O_4_ (N_2_)	–10.8 ± 0.7	–14.0 ± 0.4	–17.2 ± 0.4	–12.5 ± 0.2
ZnAl_2_O_4_ (O_2_)	–17.4 ± 0.6	–19.2 ± 0.2	–20.9 ± 0.1	–23.0 ± 0.5
MgAl_2_O_4_ (N_2_)	–17.6 ± 2.2	–20.9 ± 0.7	–23.7 ± 0.6	–13.1 ± 0.6
MgAl_2_O_4_ (O_2_)	–18.8 ± 0.8	–21.4 ± 0.2	–23.7 ± 0.2	–16.2 ± 0.4

The energy of oxygen vacancy formation is calculated with respect to the gas-phase H_2_ and H_2_O molecules with the following equation:

$$(\mathrm{fully}\;\mathrm{oxidized}\;\mathrm{surface})+H_{2}\overset{\Delta E_{\mathrm{Vo}}}{\to }\left(\mathrm{surface}\;\mathrm{with}\;\mathrm{Oxygen}\;\mathrm{Vacancy}\right)+H_{2}O$$

$$\Delta E_{\mathrm{Vo}}=\left(E_{\mathrm{surf},\mathrm{Vo}}+E_{H_{2}O}\right)-\left(E_{\mathrm{surf},\mathrm{fully oxidized}}+E_{H_{2}}\right)$$

where *E*_surf,Vo_ = energy of surface with an oxygen vacancy, $E_{H_{2}O}$ = energy of water in the gas phase, *E*_surf,fully oxidized_ = energy of fully oxidized surface, and $E_{H_{2}}$ = energy of hydrogen molecule in the gas phase.

## Results and Discussion

Spinels are a large family of minerals that are typically found in nature as oxides with the general formula AB_2_O_4_, where A and B are divalent and trivalent metal cations, respectively. The prototype spinel is magnesium aluminate MgAl_2_O_4_, which adopts the cubic *Fd*3̅*m* space group with a unit cell parameter of *a* = 8.086 Å. Its structure can be described as a cubic close-packed (ccp) array of oxygen anions with Mg^2+^ cations occupying 1/8 of the tetrahedral sites and Al^3+^ occupying 1/2 of the octahedral sites (Figure 1A). Zinc aluminate ZnAl_2_O_4_ is another prominent member of the spinel family with a similar lattice parameter (*a* = 8.085 Å) due to Zn^2+^ and Mg^2+^ having nearly identical ionic radii.^38^ Although the metal cations have similar sizes, the corresponding binary oxides have different structures due in part to differences in electronegativity. Here, magnesium oxide (MgO) adopts the cubic rocksalt-type structure (*Fm*3̅*m*; *a* = 4.212 Å), where the arrangement of edge-sharing MgO_6_ octahedra minimizes repulsive interactions between Mg^2+^ metal centers (Figure 1B). Conversely, ZnO adopts the hexagonal wurtzite-type structure (*P*6_3_ *mc*; *a* = 3.252 Å, *c* = 5.206 Å) that is composed of corner-sharing ZnO_4_ tetrahedra (Figure 1C).

**Figure 1:**
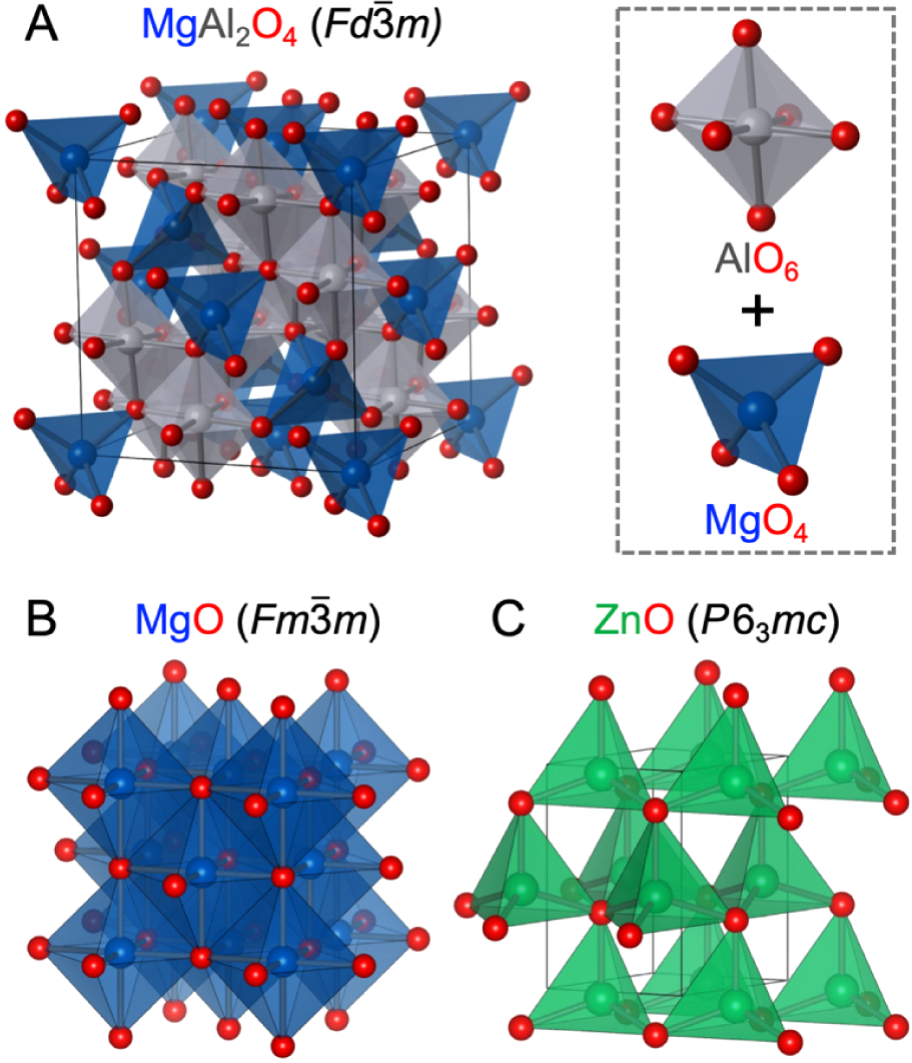
Crystal structures of ternary and binary metal oxides. (A) Spinel-type MgAl_2_O_4_ has a cubic close-packed (ccp) array of O^2–^ anions, where Mg^2+^ and Al^3+^ occupy tetrahedral and octahedral sites, respectively. (B) Rocksalt-type MgO is cubic and composed of edge-sharing MgO_6_ octahedra, and (C) wurtzite-type ZnO is hexagonal and composed of corner-sharing ZnO_4_ tetrahedra. Mg atoms are depicted in blue, Al in gray, Zn in green, and O in red.

Ternary MAl_2_O_4_ and (M = Mg^2+^, Zn^2+^) oxides were synthesized by using a hydrothermal protocol followed by high-temperature annealing under various conditions, as discussed in the Experimental Section. The products were pulverized using a mortar and pestle, and the resulting powders were structurally characterized using XRD. Figure 2 shows X-ray diffractograms for spinel products treated at 900 °C in oxygen and nitrogen atmospheres, along with the simulated XRD pattern, while XRD diffractograms of spinels annealed in air can be seen in Figure S2. In each case, the reflections in the experimental diffractograms in Figure 2A are consistent with those of spinel-type ZnAl_2_O_4_ without any observable impurities. Figure 2B shows the diffractograms of the MgAl_2_O_4_ products annealed under the same conditions. Again, the observed peaks match the simulated XRD pattern without any noticeable impurities. We note that slight peak broadening, when compared with the ZnAl_2_O_4_ patterns, can be attributed to smaller crystallites and associated microstrain in the MgAl_2_O_4_ particles. While microstrain can influence the nature of surface defects, it is challenging to correlate these features with the concentration and type of *V*_O_ on the oxide surfaces. Since the motivation was to identify broader trends across a range of systems, further inquiry was deemed outside the scope of this study. In summary, the XRD data indicate that the ternary oxides are phase-pure spinels, and the annealing atmosphere does not significantly affect the bulk crystallinity of the samples.

**Figure 2:**
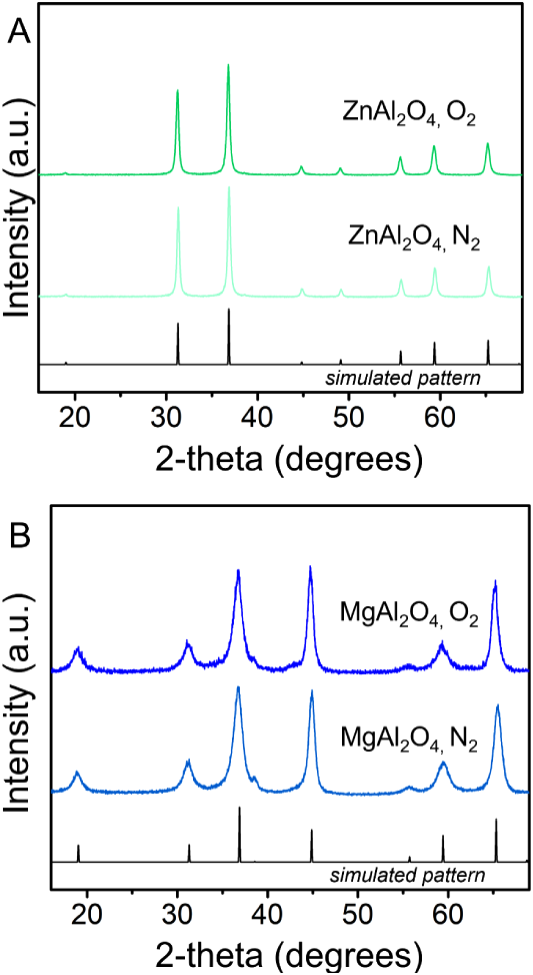
X-ray diffractograms of (A) ZnAl_2_O_4_ and (B) MgAl_2_O_4_ powders annealed in oxygen and nitrogen atmospheres. In each case, the peaks in the experimental data are consistent with the simulated patterns with no observable impurities.

Scanning electron microscopy (SEM) was used to characterize the homogeneity and morphology of the spinel oxide particles. Figure S3 shows SEM images of ZnAl_2_O_4_ and MgAl_2_O_4_ annealed under different conditions and indicates that particle size and morphology do not change substantially upon heating in different environments. For ZnAl_2_O_4_, the particles appear visually larger than the MgAl_2_O_4_ particles, with particles in the 10–25 μm range for ZnAl_2_O_4_ and 1–10 μm for MgAl_2_O_4_.

Commercially purchased MgO and ZnO binary oxides were annealed under the same conditions as for the spinel systems and structurally characterized using powder XRD. The X-ray diffractograms shown in Figure 3A indicate that each of the ZnO samples has the wurtzite-type structure and is phase pure. The XRD data for the MgO samples are shown in Figure 3B, and each pattern exhibits peaks that are consistent with the rocksalt-type MgO and have no observable impurities. The XRD diffractograms of binary oxides annealed in air can be found in Figure S4 and are consistent with the simulated patterns.

**Figure 3:**
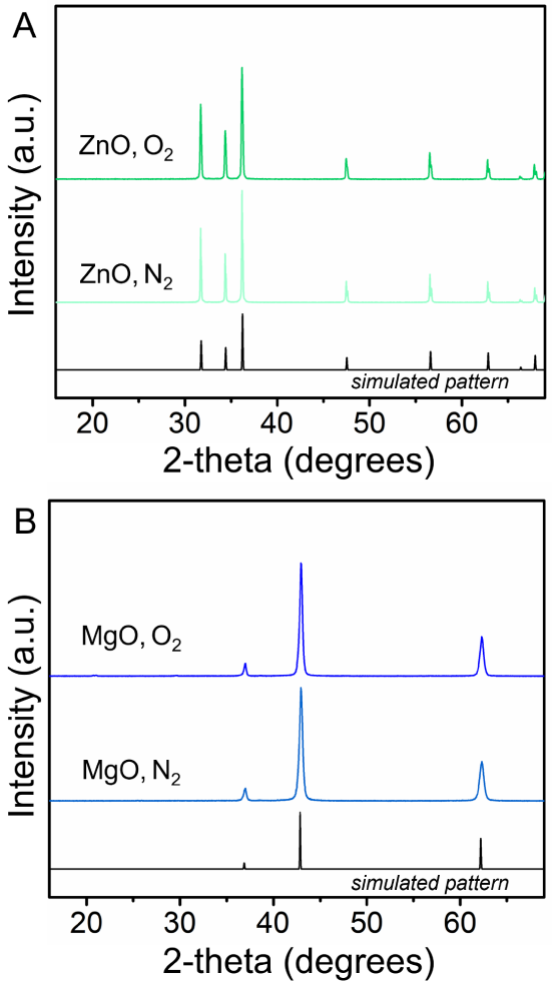
(A) XRD patterns for ZnO samples annealed in oxygen and nitrogen atmospheres, with the simulated pattern plotted below. (B) XRD patterns for MgO samples annealed in oxygen and nitrogen atmospheres along with the simulated pattern.

The surface areas of the particles of the ternary spinel and binary oxide samples (Table S5) were measured using Brunauer–Emmett–Teller (BET) methods to normalize the ice nucleation activity of the spinels and metal oxides under investigation with respect to the estimated density of available surface sites.^39^ All the spinel surface areas span the same order of magnitude, while the metal oxide surface areas vary over 1 order of magnitude.

The frozen fraction plots corresponding to ZnAl_2_O_4_ and MgAl_2_O_4_ after exposure to O_2_ and N_2_ are displayed in Figure 4A. These data illustrate the temperature-dependent ice nucleation activity of the prepared materials. Flattening of the curves during the initial or final stages of each freezing assay results from isolated instances of suspension droplets freezing at warmer or colder temperatures than the majority of droplets, respectively. Thus, it is difficult to draw direct conclusions from the interpretation of these curves. To assess the ice nucleation activity of ZnAl_2_O_4_ and MgAl_2_O_4_, one method is to report the *T*_10_, *T*_50_, and *T*_90_ values of each sample, or the temperatures at which 10, 50, and 90% of the total number of suspension droplets freeze. These temperatures are determined by fitting the frozen fraction data to a sigmoidal curve. The slope average and standard deviation were obtained by applying a linear fit to the mean *T*_10_, *T*_50_, and *T*_90_ values. Both the O_2_- and N_2_-treated spinel results are organized in Table 1 for simplicity, while the data set for the air-treated batches is provided in Table S3. While *T*_10_ for both the air-treated and N_2_-treated ZnAl_2_O_4_ do not differ significantly, a steeper slope for the latter suggests that the active sites induce ice formation more uniformly along the N_2_-treated ZnAl_2_O_4_ surface compared to the air-treated ZnAl_2_O_4_ surface. Although the slope corresponding to the O_2_-treated ZnAl_2_O_4_ data is the steepest out of all the data for this sample, suggesting the greatest uniformity of active sites, the *T*_10_, *T*_50_, and *T*_90_ values all shift to colder temperatures. On the contrary, *T*_10_, *T*_50_, and *T*_90_ for MgAl_2_O_4_ treated under all three annealing atmospheres are statistically similar. Thus, the ice nucleation activity of ZnAl_2_O_4_ is more sensitive to the type of postsynthesis treatment than MgAl_2_O_4_. The corresponding ice nucleation active site (INAS) density plot (Figure 4B) illustrates that ZnAl_2_O_4_ possesses a greater quantity of nucleation sites per unit surface area at warmer temperatures when exposed to N_2_ compared to O_2_. A similar increase in the INAS density of MgAl_2_O_4_ is observed after replacing the O_2_ with N_2_. Therefore, for both spinels, employing a reducing atmosphere enhances their ice nucleation activity at warmer temperatures. In comparing ZnAl_2_O_4_ to MgAl_2_O_4_, we observe that the ice nucleation activity for ZnAl_2_O_4_ is greater than MgAl_2_O_4_ when the same annealing atmosphere is used, though ZnAl_2_O_4_ calcined under O_2_ overlaps with MgAl_2_O_4_ calcined under N_2_.

**Figure 4:**
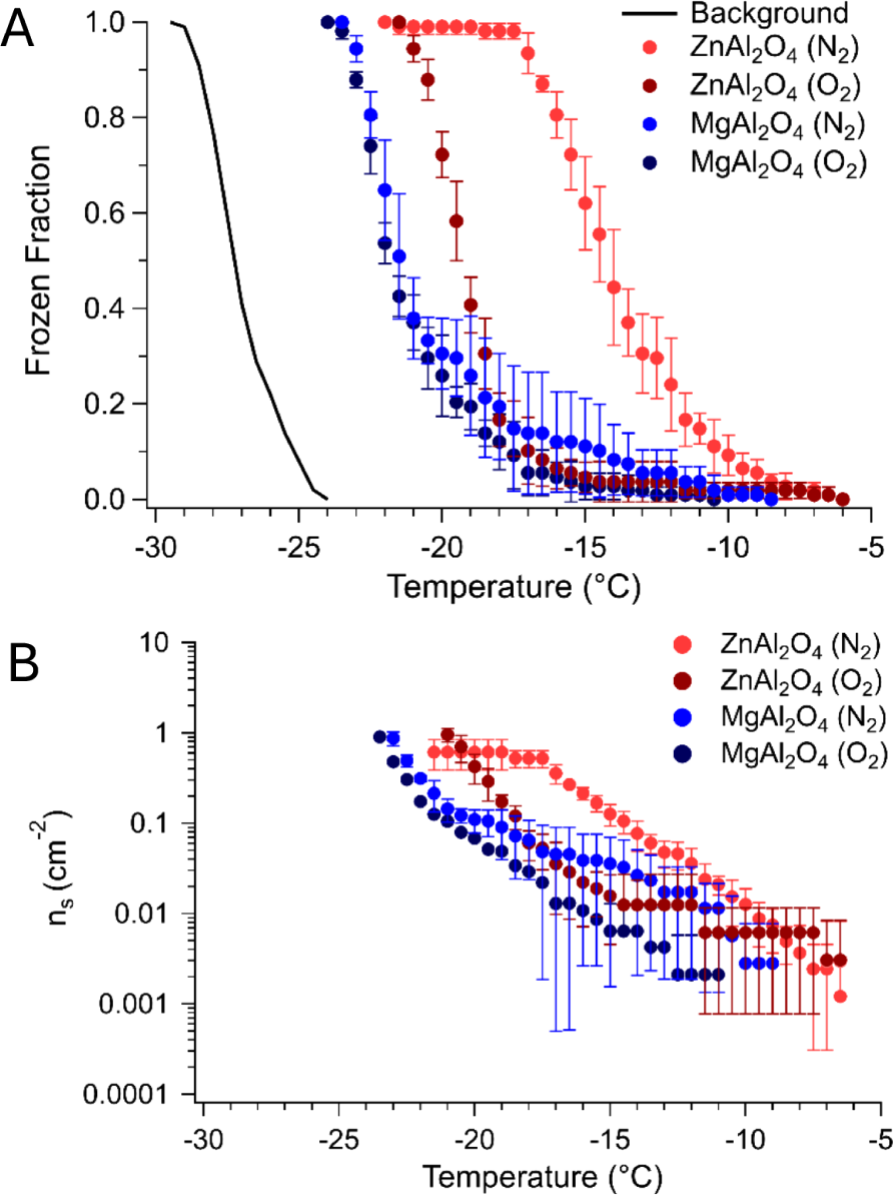
A) Frozen fraction and B) INAS density graphs relating to ZnAl_2_O_4_ and MgAl_2_O_4_ spinels after annealing in N_2_ and O_2_ atmospheres. In B), only the upper error bar, indicating the standard deviation across measurements, is displayed for select data points, while the lower error bar is not shown for these data points due to the usage of a logarithmic scale.

Regardless of the postsynthesis atmosphere, substituting Zn^2+^ for Mg^2+^ yields an increase in the INAS density, highlighting the influence of the non-Al cation (in other words, the A cation in the generic spinel formula AB_2_O_4_) on the interactions between interfacial water and the spinel surface (Figure 4B). Since this discrepancy cannot be ascribed to differences in the size of the metal ion because the lattice parameters of both spinels closely match (Table S2), the electronic structures of each spinel surface may dictate the ice-forming mechanism. Furthermore, the hydration mechanisms of both zinc and magnesium cations also differ. While magnesium cations preferentially accommodate six water molecules in their inner coordination spheres, zinc cations permit the exchange of water molecules between their inner and outer coordination spheres without incurring a significant energy penalty, resulting in a more flexible hydration environment.^40^ Additionally, the Gibbs free energy of hydration in aqueous solutions is more negative for Zn^2+^ as opposed to Mg^2+^,^41,42^ indicating that zinc ion–water interactions are more thermodynamically favorable than magnesium ion–water interactions. Soniat et al. model the charge transfer dynamics of both Zn^2+^ and Mg^2+^ in water by performing DFT calculations, concluding that Zn^2+^ ions transfer more of their electron density to the water molecules in their first hydration shell compared to Mg^2+^ ions.^43^ The flexible interactions between the zinc ions and interfacial water molecules, in addition to the magnitude of the Gibbs free energy of hydration and enhanced charge transfer, may facilitate ice nucleation on the surface of ZnAl_2_O_4_ as opposed to MgAl_2_O_4_. However, the aforementioned studies address hydration thermodynamics for free ions in aqueous solution rather than cations in ionic solids, and we will not further speculate about the energetics of each scenario. Below, we aim to identify the attributes which promote ice nucleation on the spinel surface as a result of exposure to a reducing atmosphere.

We explore how the calcination atmosphere impacts the surface and bulk chemical composition of ZnAl_2_O_4_ and MgAl_2_O_4_ by performing depth-profiling XPS on the air-treated and O_2_-treated samples. Table 2 outlines the relative atomic compositions of each sample irradiated with Mg, Al, and Zr X-ray sources, which are used to excite photoelectrons at analytical depths beneath the surface ranging from 2 to 4 nm for Mg to 5 to 7 nm for Zr. The ratio of atomic percentages, (Zn or Mg)/Al, is approximately the bulk stoichiometric ratio of 0.5 at greater depths for both types of spinels. Note that the sum of Al and Zn or Mg is not 100% due to O and C contents, though C and O are not always detected due to interferences. Both ZnAl_2_O_4_ and MgAl_2_O_4_ are more depleted in Zn and Mg relative to Al along the first 2–5 nm-thick layer of the surface than at thicknesses above 5 nm, where the chemical composition resembles that of the bulk material. In addition, Mg-containing materials retain approximately the bulk stoichiometric ratio of Mg/Al at a depth of 3–5 nm, while Zn-containing materials are more depleted of Zn at these depths. When the calcination environment is switched from air to O_2_ for both spinels, the ratio of atomic percentages at each analyzed depth does not change substantially. Therefore, we hypothesize that the ice nucleation activities of ZnAl_2_O_4_ and MgAl_2_O_4_ are predominantly influenced not by the relative compositions of Zn/Mg and Al at the surface, but rather by other heterogeneities that emerge during the annealing process.

**Table 2:** XPS Quantification of Zn and Mg to Al Ratios in Both Air-Treated and O_2_-Treated ZnAl_2_O_4_ and MgAl_2_O_4_ from Depth Profiling Experiments

		Concentration (Rel. Atom%)		
Sample	X-ray Source	Zn or Mg	Al	(Zn or Mg)/Al
ZnAl_2_O_4_ (Air)	Mg (2–4 nm)	7.4	27.5	0.27
	Al (3–5 nm)	8.3	26.4	0.31
	Zr (5–7 nm)	34.4	65.6	0.52
ZnAl_2_O_4_ (O_2_)	Mg (2–4 nm)	7.4	29.1	0.25
	Al (3–5 nm)	7.9	26.6	0.30
	Zr (5–7 nm)	33.6	66.4	0.51
MgAl_2_O_4_ (Air)	Mg (2–4 nm)	9.3	28.6	0.32
	Al (3–5 nm)	12.0	24.5	0.49
	Zr (5–7 nm)	35.3	64.7	0.55
MgAl_2_O_4_ (O_2_)	Mg (2–4 nm)	8.7	27.5	0.32
	Al (3–5 nm)	11.0	25.1	0.44
	Zr (5–7 nm)	36.5	63.5	0.57

We hypothesize that the bandgap affects how stable defects are in specific compounds. For example, refractory materials and others with high bandgaps are less susceptible to form vacancies (and other defects), whereas lower bandgap compounds can more easily accommodate these vacancies (i.e., dopants). To further probe this feature, ZnO and MgO were chosen due to their similar bandgap differences with spinels. Specifically, the bandgaps of ZnAl_2_O_4_ and MgAl_2_O_4_ are reported to be approximately 3.8 and 7.8 eV,^44^ respectively, while the bandgaps for ZnO and MgO are reported to be approximately 3.2 eV^45^ and 7.8 eV,^46^ respectively. We monitor the immersion freezing efficiency of both ZnO and MgO following treatment under all three calcination atmospheres. For simplicity, Figure 5a displays the frozen fraction plots pertaining to N_2_- and O_2_-treated ZnO and MgO, and Figure S6 contains data for ZnO and MgO treated under all three annealing atmospheres. The range of freezing temperatures for ZnO between the O_2_- and N_2_-annealed samples is broader compared to MgO treated under both atmospheres, and the N_2_- annealed binary oxides are more susceptible to ice nucleation than the O_2_-annealed binary oxides. This observation also holds true for the spinels, as described previously. In addition, *T*_10_, *T*_50_, and *T*_90_ are all warmest for ZnO and MgO after annealing in N_2_ compared to ZnO and MgO annealed under O_2_ (Table 3) and air (Table S4). Likewise, the *T*_10_ and *T*_50_ values for MgO under air and O_2_ annealing atmospheres are statistically similar. Like ZnAl_2_O_4_ and MgAl_2_O_4_, ZnO is more prone to undergoing chemical changes, such as the formation of oxygen vacancies, than MgO after being introduced to a strongly reducing atmosphere. However, in contrast to the ice nucleation data trends for spinels, MgO nucleates ice at warmer temperatures than ZnO when they are annealed with O_2_. Since ZnO possesses a wurtzite structure containing tetrahedrally coordinated Zn ions and MgO has a rock salt structure with octahedrally coordinated Mg ions, crystallographic differences are expected to influence the interactions of each metal oxide with interfacial water. However, the focus of our work involves exploring how annealing atmosphere (air, nitrogen, oxygen) influences the ice nucleation activity of a specific system. According to the INAS density data shown in Figure 5b, at warmer temperatures, the cumulative number of active sites per unit surface area increases for N_2_-treated ZnO compared to O_2_-treated ZnO, while *n*_s_ for MgO does not increase as significantly.

**Figure 5:**
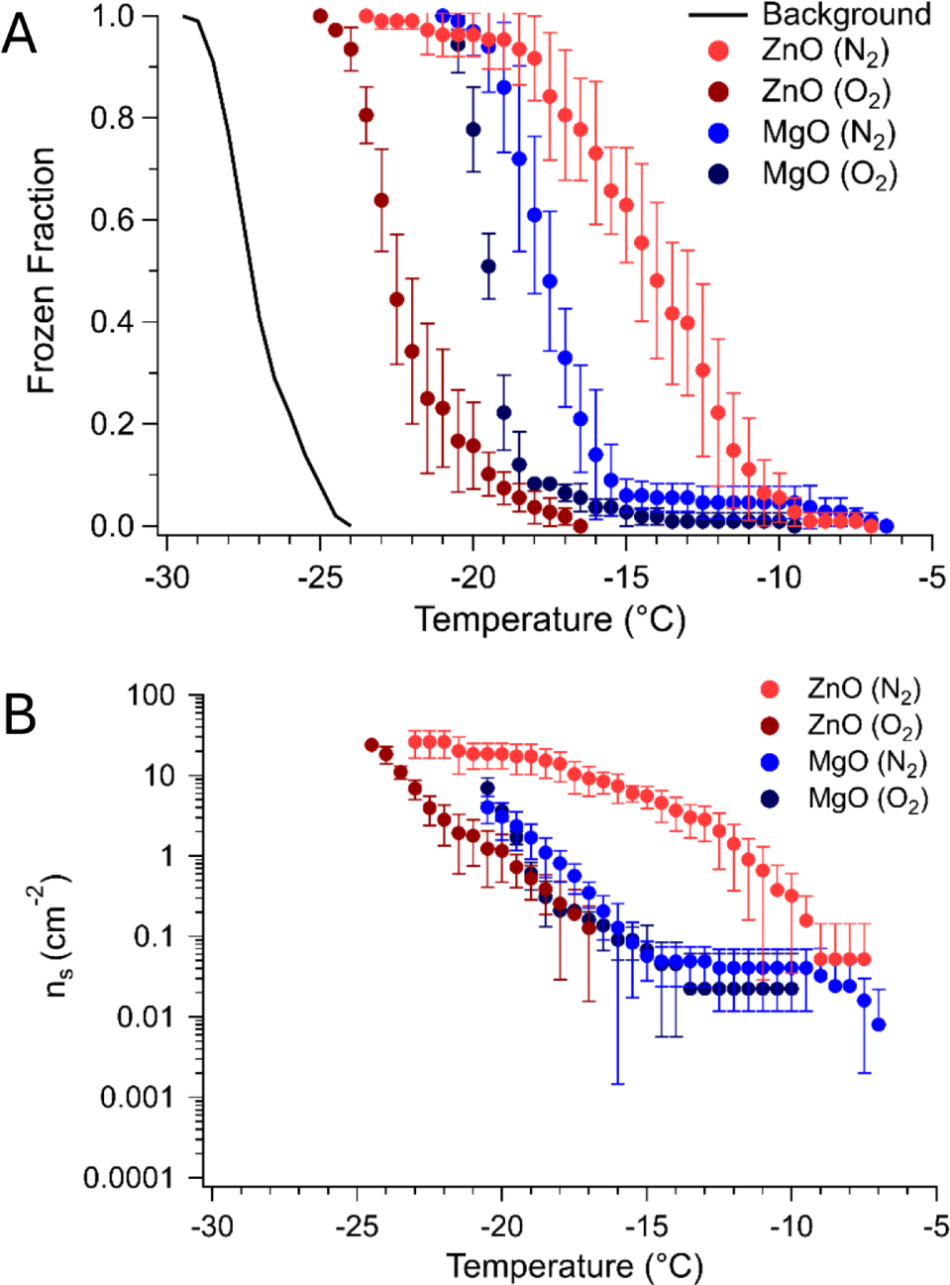
A) Frozen fraction and B) INAS density graphs relating to ZnO and MgO after annealing in N_2_ and O_2_ atmospheres. In part B, only the upper error bar, indicating the standard deviation across measurements, is displayed for select data points, while the lower error bar is not shown for these data points due to the usage of a logarithmic scale.

**Table 3:** *T*_10_, *T*_50_, and *T*_90_ Values, as well as Slopes, for ZnO and MgO Treated under N_2_ and O_2_

Sample	*T*_10_ (°C)	*T*_50_ (°C)	*T*_90_ (°C)	Slope (% frozen/°C)
ZnO (N_2_)	–10.5 ± 0.8	–14.2 ± 1.1	–18.0 ± 1.5	–10.6 ± 0.1
ZnO (O_2_)	–20.3 ± 1.1	–22.4 ± 0.6	–24.3 ± 0.1	–20.0 ± 0.3
MgO (N_2_)	–15.6 ± 0.6	–17.6 ± 0.5	–19.4 ± 0.7	–21.4 ± 0.5
MgO (O_2_)	–18.5 ± 0.3	–19.5 ± 0.1	–20.4 ± 0.2	–42.6 ± 0.6

Overall, the ice nucleation activity of both ZnO and ZnAl_2_O_4_ is more dependent on the postsynthesis calcination conditions than MgO and MgAl_2_O_4_. Specifically, annealing in an N_2_–rich atmosphere enables ice nucleation at warmer temperatures. We cannot ascribe these differences in ice nucleation activity to variations in the zinc, magnesium, and aluminum cation concentrations, as determined by XPS, because the ratios of Zn or Mg to Al are similar at the interface. Instead, we hypothesize that all of the materials tested contain oxygen vacancy point defects, *V*_O_, at their surfaces and that these features may be more prevalent and energetically favorable in the Zn-containing materials used in this study. The activity of *V*_O_ sites has been highlighted in studies addressing their effect on the wettability of ZnO surfaces,^47^ TiO_2_ nanorods,^48^ and the kinetics of water dissociation along rutile TiO_2_(110) thin films.^49^ Interestingly, oxygen vacancy sites formed from irradiation of TiO_2_(110) with electrons are found to weaken the formation of epitaxial ice on these substrates.^50^ In addition, *V*_O_ sites enhance the photoluminescence of ZnAl_2_O_4_ at higher calcination temperatures^51^ and MgAl_2_O_4_ when more rigorous annealing conditions are employed.^52^ *V*_O_ sites are prone to formation on the surface of ZnO under inert and high-temperature annealing atmospheres^53^ as well as MgO after the application of external mechanical forces to intensify its mechanoluminescence.^54^ We hypothesize that heterogeneous ice nucleation is also impacted by *V*_O_ being positioned on the surface. Although we considered quantifying the amount of *V*_O_ sites confined to the surface by conducting a chemisorption study, it would be difficult to accurately determine exact concentrations of these defect sites in this manner.

Density functional theory (DFT) calculations were employed to gain insight into the formation energies of *V*_O_ on the studied oxide surfaces. Specifically, we employed PBE, an exchange-correlation functional in DFT, to calculate the energy of oxygen vacancy formation with respect to the gas-phase H_2_ and H_2_O. Wulff constructions were computed to predict the shapes and exposed facets of the MgAl_2_O_4_, ZnAl_2_O_4_, MgO, and ZnO crystallites,^36,37^ which are shown in panels A–D of Figure 6, respectively. It is important to note that even though both spinels have the same crystal structure, their crystal shape and exposed facets can be different. This is observed in most systems when assessing the surface energy of compounds with a common structure type.^55^ DFT results show that the oxygen vacancy formation energy (*E*_Vo_) on the most dominant facet of the ZnAl_2_O_4_ crystal is more than 1.0 eV lower than *E*_Vo_ on the dominant facet of the MgAl_2_O_4_ crystal (Table 4). The weighted average *E*_Vo_ value for each spinel was calculated using the distribution of facets predicted from the Wulff constructions, showing that the *E*_Vo_ for ZnAl_2_O_4_ (0.03 eV) is more than 0.4 eV lower than MgAl_2_O_4_ (0.45 eV). The calculations indicate that surface oxygen vacancies on ZnAl_2_O_4_ are thermodynamically more favorable on their dominant facets than on MgAl_2_O_4_. Similarly, the weighted average *E*_Vo_ value for ZnO (0.90 eV) is more than 3.0 eV lower than the *E*_Vo_ value for the MgO crystal (3.92 eV) (Table 4). This correlates well with the discrepancy in the experimentally determined ice nucleation activity of the Mg- and Zn-containing samples. Specifically, the calculations suggest that ZnAl_2_O_4_ and ZnO can better facilitate ice nucleation, relative to MgAl_2_O_4_ and MgO, because it is energetically easier to create oxygen vacancies on their surfaces. Similar calculations were performed by Hinuma et al. which link the oxygen vacancy formation energies *E*_Vo_ and band gap energies of numerous metal oxides, including wurtzite ZnO and rocksalt MgO.^56^ They report that the (100) facet of ZnO possesses a band gap of 1.47 eV compared to 4.65 eV for the (100) surface of MgO, with the corresponding *E*_Vo_ values being 3.57 and 6.27 eV, respectively. Thus, *V*_O_ defects are expected to be more prevalent along the (100) facet of ZnO rather than the (100) facet of MgO. In contrast, the band gap and weighted *V*_O_ formation energies of bulk metal oxides correlate inversely with each other, as determined computationally.^57–59^

**Figure 6:**
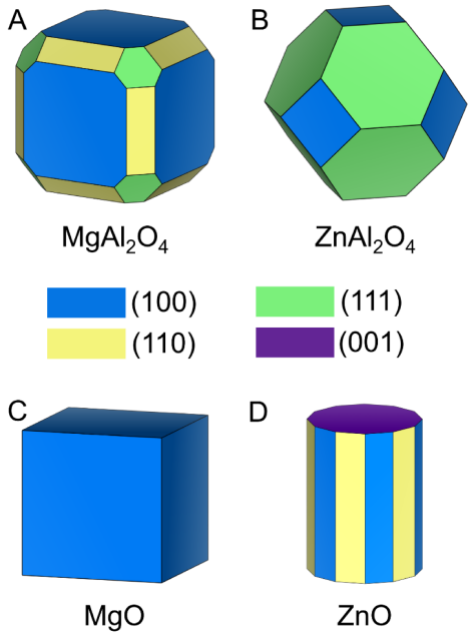
Wulff construction of A) MgAl_2_O_4_, B) ZnAl_2_O_4_, C) MgO, and D) ZnO single-crystal shapes. The (100) surface is displayed in blue, (110) in yellow, (111) in green, and (001) in dark purple.

**Table 4:** Oxygen Vacancy Formation Energies

crystal system	oxide	facet	(10–10)	(11–20)	(001)	weighted d*E*_vo_ (eV)
hexagonal	ZnO	d*E*_vo_ (eV)	0.73	0.67	1.46	0.90
		exposed ratio of facet	35.70%	37.80%	26.50%	

crystal system	oxide	facet	(100)	(110)	(111)	weighted d*E*_vo_ (eV)
cubic	MgO	d*E*_vo_ (eV)	3.92	2.83	4.31	3.92
		exposed ratio of facet	100.00%	0.00%	0.00%	
	ZnAl_2_O_4_	d*E*_vo_ (eV)	0.48	–0.73	–0.11	0.03
		exposed ratio of facet	23.36%	0.00%	76.64%	
	MgAl_2_O_4_	d*E*_vo_ (eV)	1.01	–0.9	0.22	0.45
		exposed ratio of facet	64.58%	25.44%	9.98%	

## Conclusions & Atmospheric Implications

Our study is the first to highlight the effects of oxygen vacancy sites on the ice nucleation activity of metal oxide mineral dust aerosol particles, the structures of which contain these sites as a prevalent defect. All materials, especially ZnO and ZnAl_2_O_4_, exhibited enhanced ice nucleation activity when exposed to N_2_ gas as opposed to O_2_ and air, highlighting the influence of both cation characteristics and the formation of oxygen vacancy sites during treatment. We confirmed via XPS that the calcination conditions minimally impact the bulk and surface composition with respect to the inorganic atoms and instead hypothesize that *V*_O_ defects promote ice nucleation. According to our DFT results, ZnAl_2_O_4_ and ZnO are more favorable to accommodate ice compared to MgAl_2_O_4_ and MgO because it is energetically easier to create oxygen vacancies on the ZnAl_2_O_4_ and ZnO crystal surfaces. Given that materials in the atmosphere undergo physical processes such as weathering and oxidation, controlling oxygen vacancies is a feasible strategy with which to assess the relationship between the chemical reactivity of mineral dust aerosol particles and their ice nucleation activity.

Mineral dust aerosol particles undergo weathering and redox reactions as they are transported, leading to chemical transformations of the surface material. While these systems exhibit physical defects such as cracks and steps and may nucleate ice efficiently at these features, we have demonstrated that point defects, caused by oxygen vacancies in this case, also affect their ice nucleation activity. Specifically, we conclude that oxygen vacancy sites shift the ice nucleation activity of minerals to warmer temperatures. These sites may be present in the metal oxide components of mineral dust aerosol particles, impacting their ice nucleation activity and thus potentially affecting the microphysical properties of clouds.

## Supplementary Material



## Notes

Schematic of the immersion freezing chamber; XRD and SEM characterization, frozen fraction, and *n*_s_ data for ZnAl_2_O_4_, MgAl_2_O_4_, ZnO, and MgO treated at 900 °C under air, O_2_, and N_2_ atmospheres; *T*_10_, *T*_50_, and *T*_90_ values for air-treated spinels and metal oxides; parameters for DFT calculations; and BET data (PDF)
