# Supplementary material for: Immersion Freezing Efficiency of ZnAl2O4 and MgAl2O4 Spinels, ZnO, and MgO: The Role of Oxygen Vacancies
Source: J Phys Chem A. 2026 Feb 9;130(7):1524–33. doi: 10.1021/acs.jpca.5c06327 (PMC12927014; doi:10.1021/acs.jpca.5c06327)
Supplement: Supplementary file 1 [file jp5c06327_si_001.pdf]

Supporting Information for

Immersion Freezing Efficiency of $ZnAl_2O_4$ and $MgAl_2O_4$ Spinels, ZnO, and MgO: The Role of Oxygen Vacancies

Ryan Mitch[1,#], Ayat Tassanov[1,#], Brendan P. Troesch[1], Mikyung Hwang[2], Nathan Baumann[1], Konstantinos Alexopoulos[2], James M. Hodges[1,*], Miriam Arak Freedman[1,3,*]

[1] Department of Chemistry, The Pennsylvania State University, University Park, Pennsylvania 16802, United States

[2] Department of Chemical Engineering, The Pennsylvania State University, University Park, Pennsylvania 16802, United States

[3] Department of Meteorology and Atmospheric Sciences, The Pennsylvania State University, University Park, Pennsylvania 16802, United States

[#] These authors have contributed equally to this work.

[*] To whom all correspondence should be addressed: Miriam Freedman, maf43@psu.edu; James Hodges, hodges@psu.edu

Summary: This Supporting Information contains a diagram of the immersion freezing chamber in addition to frozen fraction, BET, and n_s data for $ZnAl_2O_4$, $MgAl_2O_4$, ZnO, and MgO treated at 900°C under air, O_2, and N_2 atmospheres. In addition, the X-ray diffractograms of all samples annealed under air are included as well as scanning electron microscopy images of the spinel samples. In addition, T_{10}, T_{50}, and T_{90} values for the air-treated spinels and metal oxides and information on DFT calculations are included.

Immersion Freezing Chamber

The diagram shown below illustrates the chamber used to conduct the droplet freezing assay experiments. In brief, high-purity N_2 gas is directed through a copper coil submerged in liquid nitrogen before entering a copper cold stage, on which a slide containing droplets is placed. The droplets contain a suspension of supermicron particles of the mineral powder of interest. Temperature readings were acquired with a K-type thermocouple with a response time of 0.25 seconds.

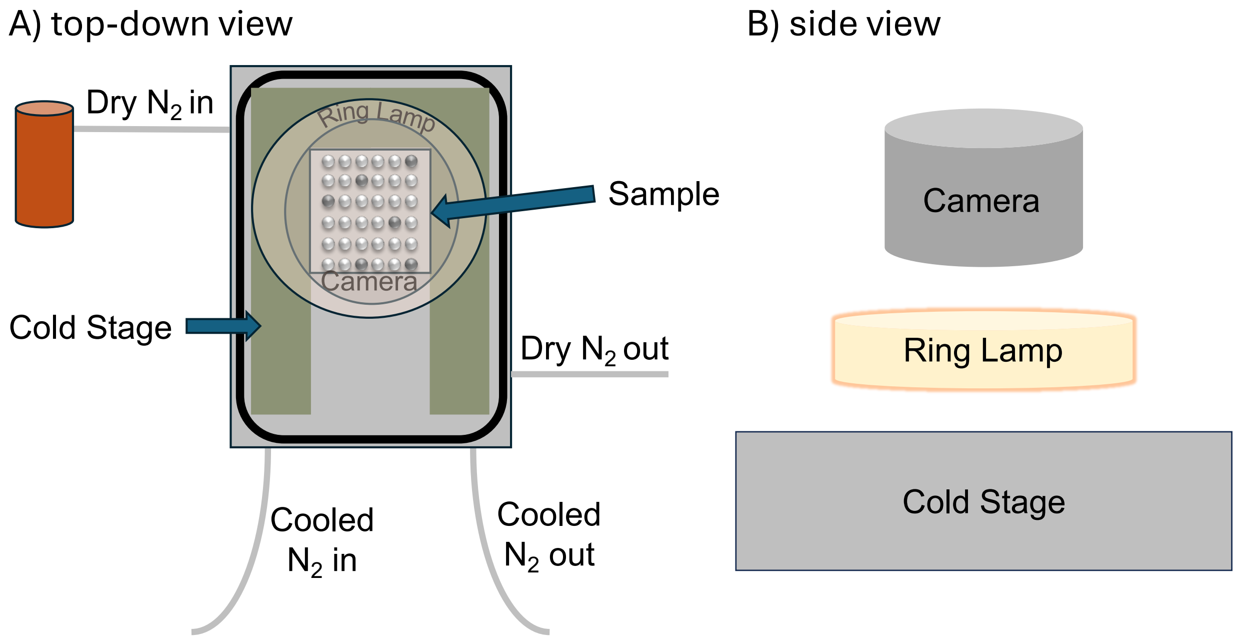

Figure S1. Schematic of the A) top view and B) right side view of the immersion freezing chamber. Only the chamber exterior, ring lamp, and camera are included in B for simplicity.

Characterization of Samples with XRD and SEM

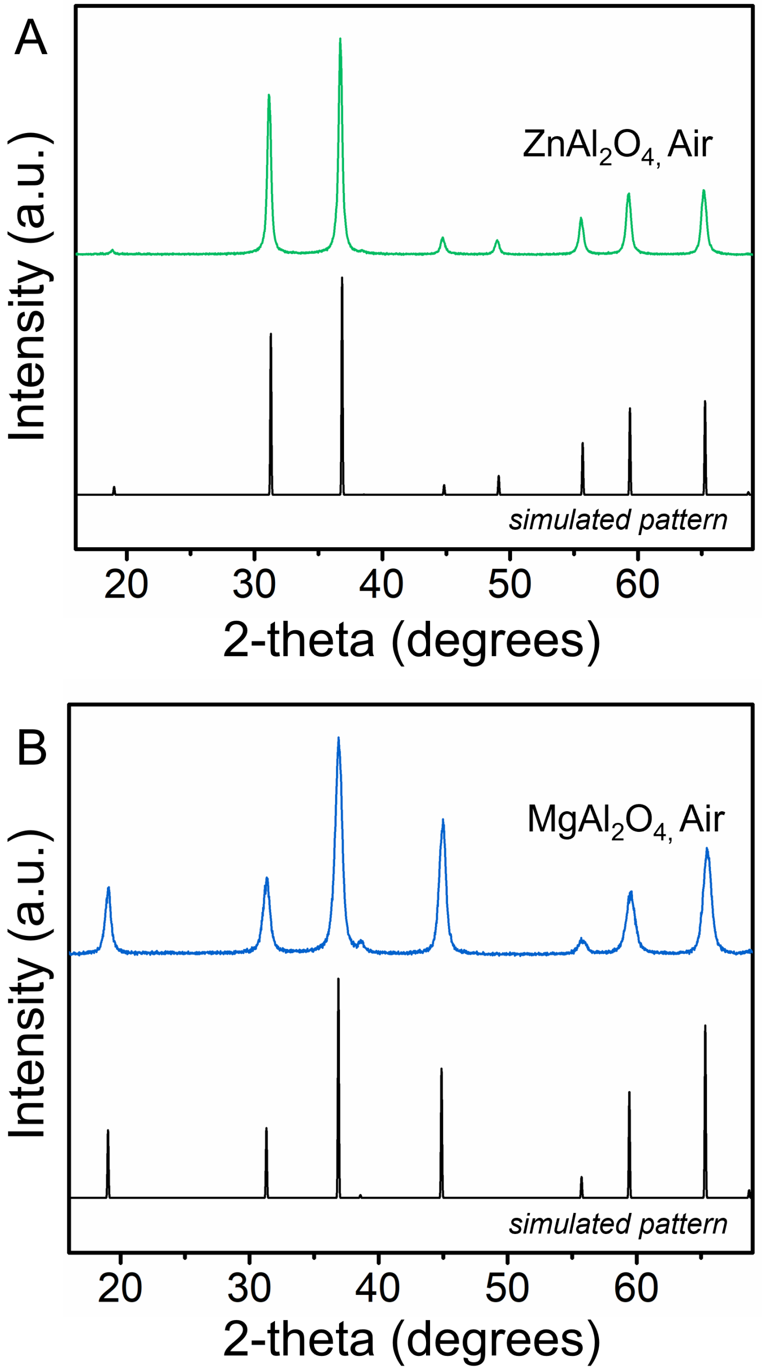

Figure S2. X-ray diffractograms of (A) $ZnAl_2O_4$ and (B) $MgAl_2O_4$ powders annealed in air. In each case, the peaks in the experimental data are consistent with simulated patterns with no observable impurities.

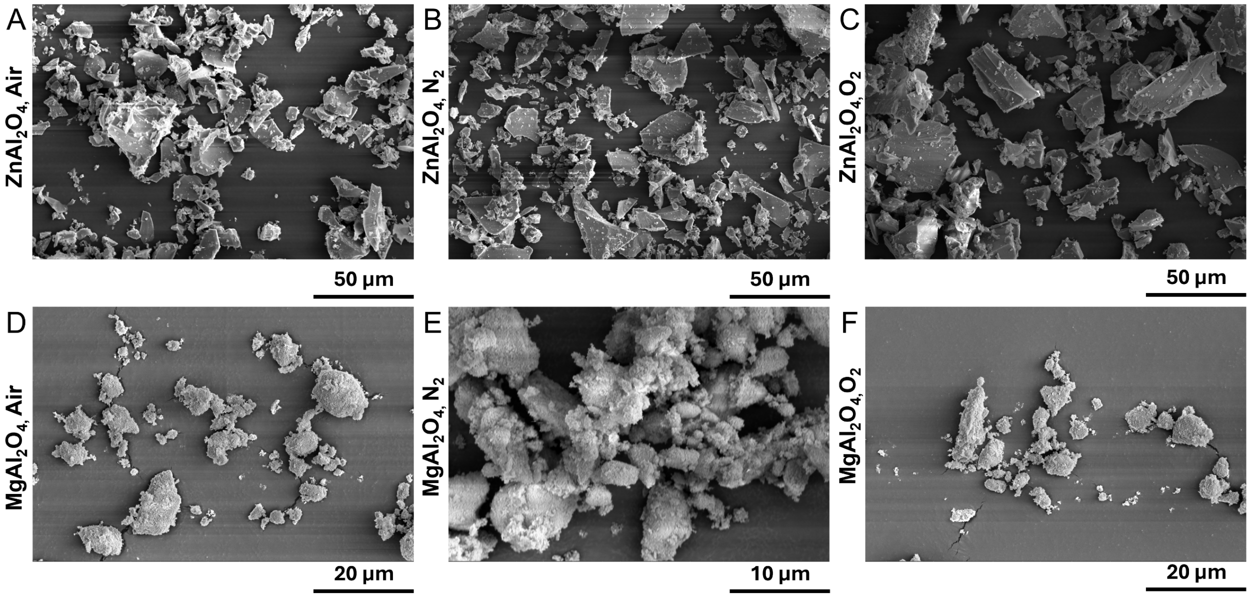

Figure S3. SEM images of (A-C) $ZnAl_2O_4$ and (D-F) $MgAl_2O_4$ powders annealed in air, nitrogen and oxygen, respectively. Both samples show no significant morphology or size change upon annealing under different atmospheric conditions.

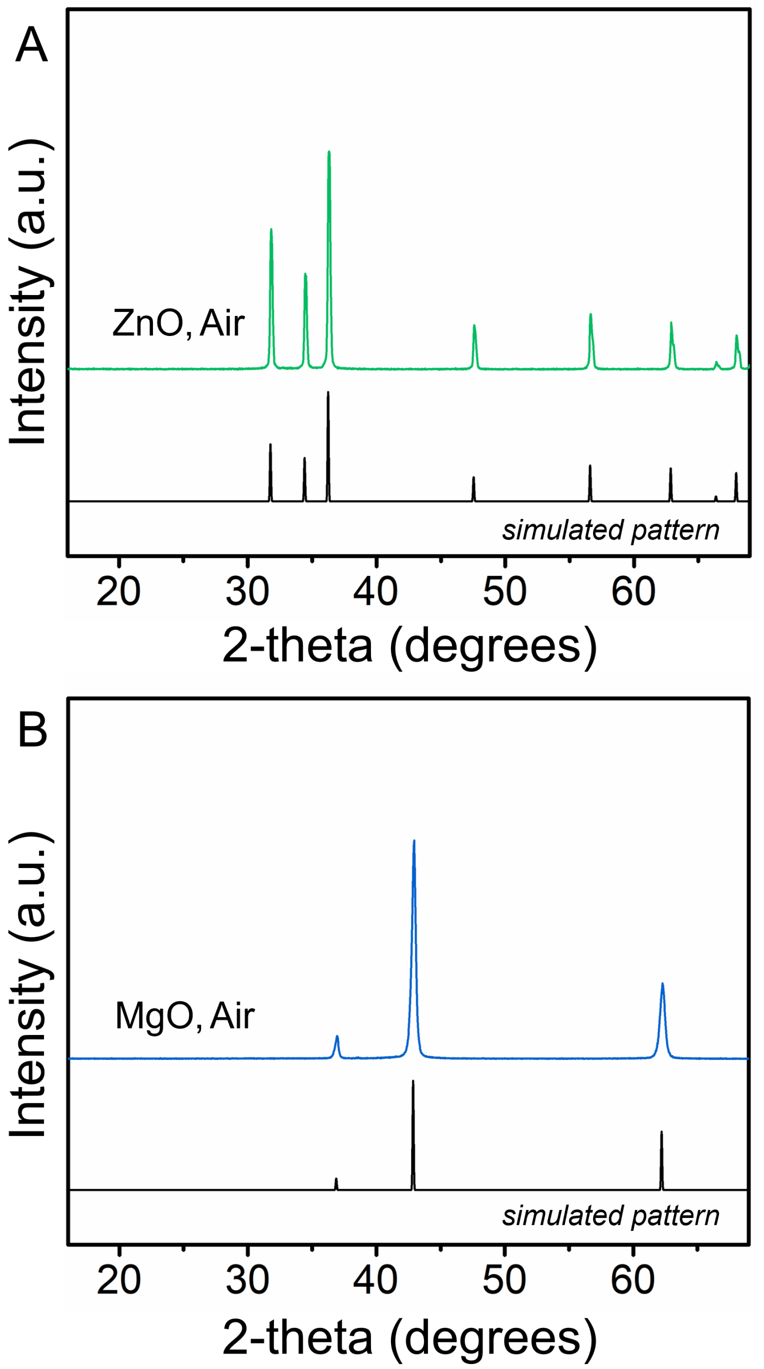

Figure S4. X-ray diffractograms of (A) ZnO and (B) MgO powders annealed in air. In each case, the peaks in the experimental data are consistent with simulated patterns with no observable impurities.

Ice Nucleation for Samples Annealed Under Air, N_2, and O_2

The graphs shown below display both frozen fraction and INAS density trends for $ZnAl_2O_4$ and $MgAl_2O_4$ annealed under air, N_2, and O_2. In (B), only the upper error bar, indicating the standard deviation across measurements, is displayed for select data points, while the lower error bar is not shown due to the usage of a logarithmic scale on the y-axis. The lower error bars are equal in magnitude to the upper error bars.

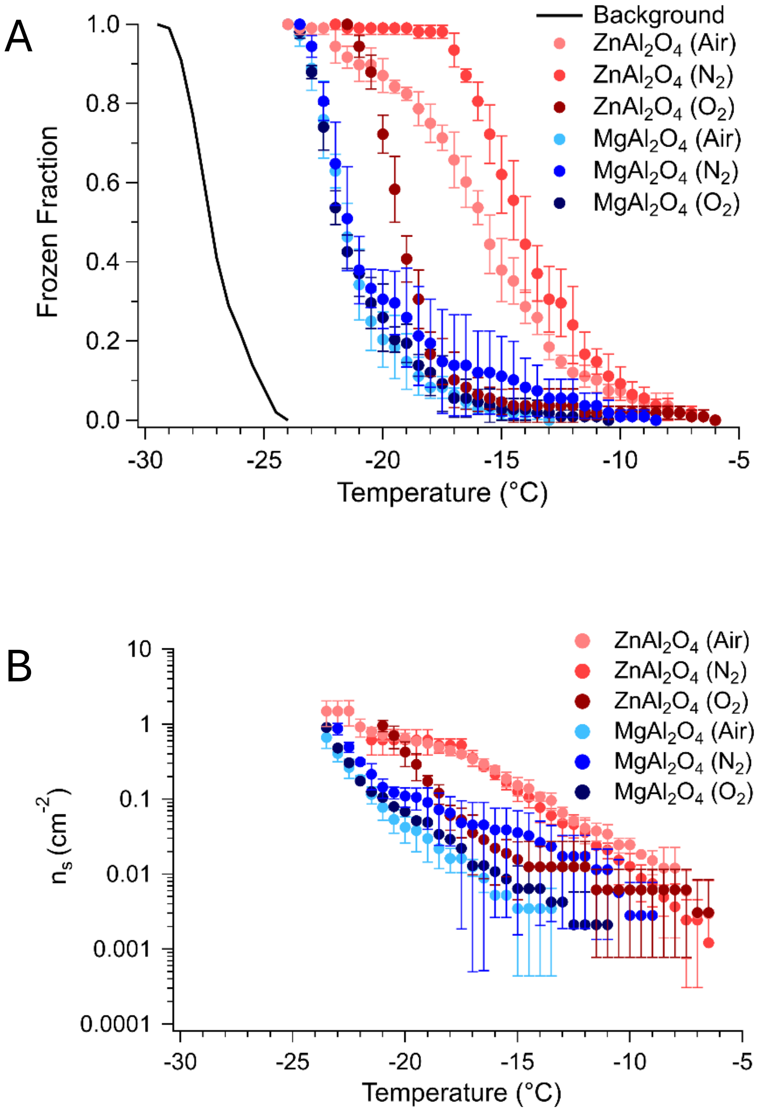

Figure S5. Summary of the A) frozen fraction and B) INAS densities of $ZnAl_2O_4$ and $MgAl_2O_4$ spinels after post-synthesis calcination at 900°C under air, N_2, and O_2 atmospheres.

The graphs shown below display both frozen fraction and INAS density trends for ZnO and MgO annealed under air, N_2, and O_2. In (B), only the upper error bar, indicating the standard deviation across measurements, is displayed for select data points, while the lower error bar is not shown due to the usage of a logarithmic scale on the y-axis. The lower error bars are equal in magnitude to the upper error bars.

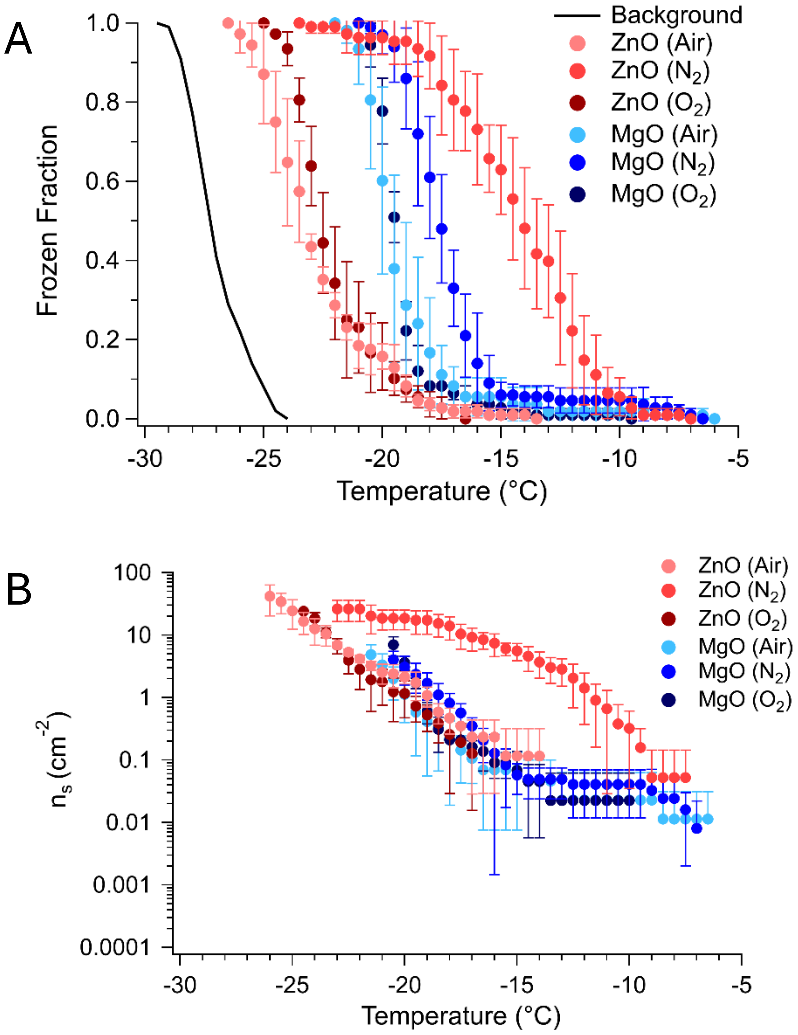

Figure S6. Summary of the A) temperature-dependent frozen fraction and B) estimated INAS densities of ZnO and MgO after post-synthesis calcination at 900°C under air, N_2, and O_2 atmospheres.

DFT Calculations of Oxygen Vacancy Formation and Spinel Lattice Parameters

DFT parameters used to compute the energetics of oxygen vacancy formation on the considered low-index facets of the metal oxides and spinels are listed in Table S1. The lattice parameters of the spinels are included in Table S2.

Table S1. Monkhorst-Pack k-point mesh and plane-wave basis set cutoff energy used for each metal oxide and low-index facet considered in developing surface models.

Metal Oxide	ZnO	MgO	$ZnAl_2O_4$	$MgAl_2O_4$
Monkhorst-Pack k-point mesh	6x6x4	5x5x5	3x3x3	4x4x4
Plane-wave basis set cutoff energy (eV)	500	400	500	500
Considered low-index facet	(100), (110), (001)	(100), (110), (111)		

Table S2. Lattice Parameters for $ZnAl_2O_4$[1]and $MgAl_2O_4$[2]

Compound	a	b	c
$ZnAl_2O_4$	8.085	8.085	8.085
$MgAl_2O_4$	8.086	8.086	8.086

Tabulated Frozen Fraction Data for Spinels and Oxides

The table shown below displays T_{10}, T_{50}, and T_{90} values for $ZnAl_2O_4$ and $MgAl_2O_4$ annealed under air. Each temperature mean and standard deviation were calculated from fitting the data to a sigmoidal curve, while the slopes were calculated by applying a linear fit to the calculated mean T_{10}, T_{50}, and T_{90}.

Table S3. T_{10}, T_{50}, and T_{90} values, as well as slopes, for $ZnAl_2O_4$ and $MgAl_2O_4$ spinels following annealing under air.

Sample	T_{10} (°C)	T_{50} (°C)	T_{90} (°C)	Slope (% frozen/°C)
$ZnAl_2O_4$ (Air)	-11.3 ± 0.7	-15.8 ± 0.4	-20.3 ± 0.1	-8.9 ± 0.0
$MgAl_2O_4$ (Air)	-19.2 ± 0.7	-21.4 ± 0.4	-23.5 ± 0.1	-19.0 ± 0.4

The table shown below displays T_{10}, T_{50}, and T_{90} values for ZnO and MgO annealed under air. Each temperature mean and standard deviation were calculated from fitting the data to a sigmoidal curve, while the slopes were calculated by applying a linear fit to the calculated mean T_{10}, T_{50}, and T_{90}.

Table S4. T_{10}, T_{50}, and T_{90} values, as well as slopes, for ZnO and MgO following annealing under air.

Sample	T_{10} (°C)	T_{50} (°C)	T_{90} (°C)	Slope (% frozen/°C)
ZnO (Air)	-20.2 ± 0.6	-23.0 ± 0.3	-25.7 ± 0.9	-14.5 ± 0.3
MgO (Air)	-17.9 ± 1.1	-19.6 ± 0.7	-21.2 ± 0.4	-24.4 ± 0.6

BET Analysis of Spinels and Metal Oxide Samples

Table S5. The BET analysis of the binary and spinel metal oxide samples.

Sample (Treatment)	BET Surface Area (m^2/g)
$ZnAl_2O_4$ (Air)	39.17
$ZnAl_2O_4$ (N_2)	95.54
$ZnAl_2O_4$ (O_2)	37.73
$MgAl_2O_4$ (Air)	67.11
$MgAl_2O_4$ (N_2)	41.29
$MgAl_2O_4$ (O_2)	54.90
ZnO (Air)	1.00
ZnO (N_2)	2.22
ZnO (O_2)	1.83
MgO (Air)	10.25
MgO (N_2)	14.53
MgO (O_2)	5.17

References

1. Holgersson, S. Lunds Universitets Arsskrift. *Avdelningen 2: Kungliga Fysiografiska Saellskapets i Lung Handlingar* **1927**, *23*, 1–9.

2. Bragg, W. The Structure of Magnetite and the Spinels. *Nature* **1915**, *95*, 561.
